# Progress in Surgery

**Published:** 1898-07-16

**Authors:** 


### Progress in Surgery.

### THE SURGERY OF THE VERMIFORM APPENDIX.

Deaver[1] contributes a paper dealing with the pathology, symptoms, and treatment of 200 cases of this condition. A careful research into the manner of invasion of the micro-organisms has clearly demonstrated that they escape into and through the wall of the appendix on account of erosion of the mucous membrane. Of 200 appendices examined, there was erosion of the mucous membrane in 149. That fæcal concretions and débris are in many cases responsible for this change there is no doubt, as in 149 there were found erosions from fæcal concretions; in 129 there was débris; and in 74 there were both—débris composed of mucus, epithelium, and degenerated mucus. When erosion occurs, these organisms have a free outlet and become at once active, causing acute inflammation of the walls of the appendix, due to the emigration, multiplication, and destructive action of the bacteria and their ptomaines. The same predominant type of micro-organism in each instance was found to be in the lumen of the appendix, in its wall, and in the lymph and pus. The most commonly found of these was the bacillus coli communis. Streptococci and staphylococci were also found in a certain percentage of cases to be the exciting factors in the production of the attack. The serum test for the colon bacillus has also been employed, according to the Widal reaction for typhoid fever. The substitution of the bacillus coli communis was made in place of that of typhoid. The reaction of the blood from cases of septic appendicitis produced by the bacillus coli communis immediately showed arrest of motility and clumping of the colon bacillus. The following table gives a good summary of the various symptoms and pathological conditions in 138 male and 62 female cases:—

| | |
|---|---|
| Constipation, 113 } 3 both. | Operation between attacks, 13. |
| Diarrhœa, 31 } 3 both. | Peritonitis, 31. |
| Normal bowels, 17. | Adhesions, 84. |
| Digestive disturbance, 45. | Cæcum or bowel involvement, 32. |
| Vomiting, 131. | Pus free in abdominal cavity, 21. |
| Operation during attack, 87. | Death, 17. |
| Gangrene, 22. | Erosion of mucous coat, 149 } 98 both. |
| Perforation, 39. | Hypertrophy, 131 } 98 both. |
| Recovery, 189. | Complete occlusion, 13. |
| Concretions, 89 } 74 both. | |
| Débris, 129 } 74 both. | |
| Partial occlusion, 50. | |
| Abscess, 59. | |
| Operation after attack, 100. | |

Barton[2] refers to 22 cases in which he had operated without removing the appendix, and they all recovered; but two of them, having a return of the disease, recovered after the removal of the appendix at the second operation. Piard[3] says the removal of the appendix in all cases increases the gravity of the operation. In the presence of suppuration situated at a distance from the appendix, the indication for removal is only absolute in the following cases: (1) When a perforating appendicitis has produced a cystic peritonitis of multiple foci. (2) When the inflamed appendix threatens to become the origin of a general septicæmia. (3) When the appendix keeps up and renews the infection in the foci at a distance. In all other cases the resection of the appendix is less urgent. The author thus sums up his study of appendicitis: (1) Abscesses are observed at a distance from the primary focus of infection in cases of appendicitis. (2) They are situated in various regions, some of which are the iliac cellular tissue, the peritoneal cavity, in the anterior abdominal parietes, in the liver, in the pleura, in the lung, and in various organs situated at greater distances, as the brain, parotid glands, kidney, and spleen. (3) These abscesses are peculiar in that they do not infect surrounding tissues, even when at a distance from the appendix, and are thus readily differentiated from peri-appendicular abscesses in abnormal situations, due to a vicious situation of the appendix. (4) These abscesses are the expression of a diffuse appendicular infection, produced by means of a peritoneal contamination, sometimes through the channel of the vascular system of the appendix by its connections, physiological or pathological. (5) They present in their variety all the transitional stages between a local inflammatory process and general septicæmia. They permit us to conceive of a new chapter to add to the auto-infections of internal origin. (6) These abscesses are rare. Their recognition will result in a more precise knowledge of the indications for operation in the surgical treatment of appendicitis. Gilbert Barling[4] publishes a list of 23 cases of internal operations in appendicitis, chiefly for relapse, all of which recovered. In reference to 12 consecutive cases of acute general peritonitis from infective appendicitis, Gould[5] makes the following observations: (1) That there is considerable variation in the clinical course of these cases. The onset may be abrupt or more gradual and remittent; the symptoms may be most severe and rapidly fatal, or for a time so little marked as to deceive any but the most careful observer; fever may be high or altogether absent; vomiting may be the most prominent feature of one case and altogether wanting in another; intestinal peristalsis may be entirely arrested or diarrhœa may exist. (2) That there is just as marked a difference in the pathological course of events. An inflamed, sloughy, and perforated appendix may be surrounded by peritoneum which, to the naked eye, appears absolutely healthy, or such an appendix may be bathed in a small quantity of turbid, dark, stinking fluid, the remainder of the sac appearing normal. In other cases there is an abundant sero-purulent exudation throughout the peritoneal cavity, or the serous membrane may be more or less widely smeared with buttery lymph; and lastly, around the diseased appendix there may be a quantity of pus indistinguishable from the thick, offensive pus of appendicular abscess, but unlimited by adhesions. (3) The urgent importance of early diagnosis and immediate operation, and the hopefulness of early operation. Of these 12 cases, all of which were operated upon, seven recovered. The author thinks that the mode of cleansing the peritoneal cavity should be varied with the conditions found at the operation. If the effusion is limited to the immediate neighbourhood of the appendix, careful and gentle wiping with a sponge is the best means to employ, but where a more general effusion is found a thorough flushing out of the cavity with a sterilised and non-irritating fluid is the best method. A drainage tube is preferable to a gauze tampon, and glass tubes are only superior to rubber tubes in those cases in which the pelvis has to be drained. Richelot[6] thinks it is well established that after the spontaneous disappearance of the symptoms of the appendix, or its surgical removal, there may still be pains which simulate those of appendicular inflammation, and that in such an event surgical intervention for the mere breakdown of adhesions is useful. Skene,[7] finding that the treatment of the pedicle of ovarian tumours with compression and heat applied with the electric current gives the best results, has employed the same method in appendicectomy with equally fortunate and gratifying results. He states that exudation from the stump and consequent pain are less after the use of the hæmostatic forceps. Deaver[8] insists on the necessity for prompt surgical interference in typhoid fever complicated by appendicitis. Under these circumstances the symptoms are not unlike those seen in appendicitis when it is present as an independent affection. Horwitz[9] relates four cases of secondary syphilis complicated with chronic appendicitis, in which a continuous course of tonic doses of mercury resulted in marked subsidence of the appendicular symptoms. All these cases, he says, were so well marked that any surgeon would have considered himself justified in operating.

[1] Annals of Surgery, Mar., 1893, p. 303. [2] Ibid., Jan., 1893, p. 103. [3] Amer. Jour. Med. Sci., Mar., 1898, p. 347. [4] Brit. Med. Jour., Jan. 29, 1898, p. 292. [5] Lancet, Jan. 1, 1898, p. 9. [6] New York Med. Jour., Mar. 12, 1893, p. 363. [7] Ibid., Mar. 5, 1898, p. 309. [8] Amer. Jour. Med. Sci., Feb., 1898, p. 189. [9] Annals of Surgery, Jan., 1898, p. 74.

### ORTHOPÆDIC SURGERY.

(*Concluded from page* 224.)

**Spondylolisthesis.**—R. W. Lovett.[24] This subject has been fully investigated by Neugebauer. In 1890 there were 101 clinical and anatomical observations. Since that time 24 have been added. The pathological condition is fairly constant; the essential part of the deformity seems to be the slipping forward of one of the lower lumbar vertebral bodies, while the vertebral arches remain practically in place. This implies, of course, an increase in the distance between the body and spinous process of such a vertebra, and the cause of this elongation or division of the vertebral arch is the subject under discussion. The commonest form of the displacement is subluxation of the fifth lumbar vertebra in its relation to the sacrum. This exists in 30 out of 42 specimens. The displacement of the fourth lumbar vertebra in relation to the fifth is next in frequency, 12 in 42 specimens. The displacement forward of the first sacral vertebra in relation to the rest of the sacrum has been recorded once only. The amount of the displacement obviously varies within very wide limits, and with it the clinical phenomena must vary also. The posterior superior iliac spines are more widely separated than normal in severe cases, and the inclination of the pelvis is diminished. The essential point of spondylolisthesis is not so much subluxation of the body of the vertebra as the antero-posterior elongation of the body of the fifth lumbar vertebra. The body may sometimes become separated from its neural arch. The causes of this are either defect in development, fracture, or primary disease of the sacro-vertebral articulation or bony changes, the result of pressure. The symptoms by which the diagnosis must be made are as follows: A disturbance of equilibrium resulting in a faulty carriage, which is shown chiefly by a sharp increase in the lower lumbar curve in even the mildest cases. More exactly it seems to be a prominence of the iliac crests and buttocks in relation to the lumbar spine. There is no apparent falling away of one spinous process from another. The spine curves forward sharply from the sacrum, and this gives undue backward prominence to the crest of the ilium and the buttocks. The appearance at first glance is the same as that in cases of double congenital dislocation of the hip. Lateral deviation of the spine may be present, and is generally indicative of a lesion more or less unilateral. With the lordosis there is a diminution of the obliquity of the pelvis, which rotates on its transverse axis. The pubis is higher than it should be normally, while the sacrum is lower. The combination of lordosis, with diminished pelvic obliquity, is said to be pathognomonic. The rotation of the pelvis is an important factor, in that it tightens the anterior ligaments of the hip and thus tends to cause a flexed position of the thighs.

**Coxa Vara Congenita.**—Kredel[25] reports two cases which he believes are of a type which he classifies as congenital. These cases are rare, and are therefore interesting. The cases present all the symptoms of coxa vara, involving both hips and being combined with other malformation of the limbs, namely, genu valgum and a severe pes equino-varus. The first case was not seen by the writer until the patient was three years old; and not until he saw the second case, only five months old, did he realise that the deformity was truly congenital. In this second case there was a unilateral coxa vara, with slight genu valgum and a severe pes equino-varus; while in the other limb there were a malformation of the knee-joint with absence of the patella, marked genu valgum, and pes equino-varus. The position preferably assumed by the child showed that the deformity was of intra-uterine origin, and due to a want of space. The limbs lay parallel, but the sound limb was abducted, while the deformed limb was adducted. The position so constantly maintained showed that this had been the position in intrauterine life. The case differed from the deformity seen in rachitic children, in that there was marked adduction present, which is said to be absent in those cases. On this point we venture to doubt. These cases showed that a deformity in this joint may originate *in utero*, and give rise to coxa vara in the early life of the child.

De Quervain thus summarises our present knowledge of this deformity. Several varieties have been described: Congenital, infantile, adolescent, and adult. The deformity is more common than is thought, and males are affected more often than females. The causes of the trouble are: In infancy, rickets; in adolescence, late rickets; and in adults, osteo-malacia. The predisposing causes are employments necessitating much standing and lifting weights. The symptoms are an insidious onset with pain in the hip and knee and limping. There is then some stiffness in the joint. The signs noticed are projection of the great trochanter, with a depression between it and the glutei, atrophy of the thigh muscles, limitation of abduction of the hip, and marked tendency to adduction. The pelvis is tilted towards the affected side, and the trochante is above Nélaton's line; in all cases the prognosis depends upon the age. Before five years of age, if the deformity is due to rickets, it may disappear spontaneously, but after this age special treatment is necessary. The affection should be diagnosed from: (1) Forward dislocation of the head of the femur; (2) recent fracture of the neck; (3) old fracture with resulting deformity; (4) separation of the epiphysis; (5) congenital dislocation; (6) coxitis due to tubercle. In the latter case the chief point is that though in early hip disease there may be slight external rotation, this is accompanied by abduction and flexion, instead of as in coxa vara adduction without flexion. The treatment is that of any constitutional disease such as rickets, and in advancing cases rest in bed with extension and abduction. But if the case has gone on for some time, and the difficulty of walking is great, an osteotomy of the neck of the femur is advisable.

[24] New York Med. Jour,, Aug. 21, 1897. [25] Amer. Jour. of Med. Sci., May, 1897. [26] La Semaine Medicale, Jan. 29, 1898.

